# Titration of Cu(I) Sites in Cu-ZSM-5 by Volumetric CO Adsorption

**DOI:** 10.1021/acsami.2c03370

**Published:** 2022-04-28

**Authors:** Gabriele Deplano, Matteo Signorile, Valentina Crocellà, Natale Gabriele Porcaro, Cesare Atzori, Bjørn Gading Solemsli, Stian Svelle, Silvia Bordiga

**Affiliations:** † Department of Chemistry, NIS and INSTM Reference Centre, Università di Torino, Via P. Giuria 7-10125 and Via G. Quarello 15/A, 10135 Torino, TO, Italy; ‡ SMN Centre for Materials Science and Nanotechnology, Department of Chemistry, University of Oslo, P.O. Box 1033, Blindern, N-0315 Oslo, NO, Norway

**Keywords:** Cu-ZSM-5, zeolites, redox speciation, Cu(I) titration, carbon monoxide, adsorption volumetry, Cu(I) monocarbonyls

## Abstract

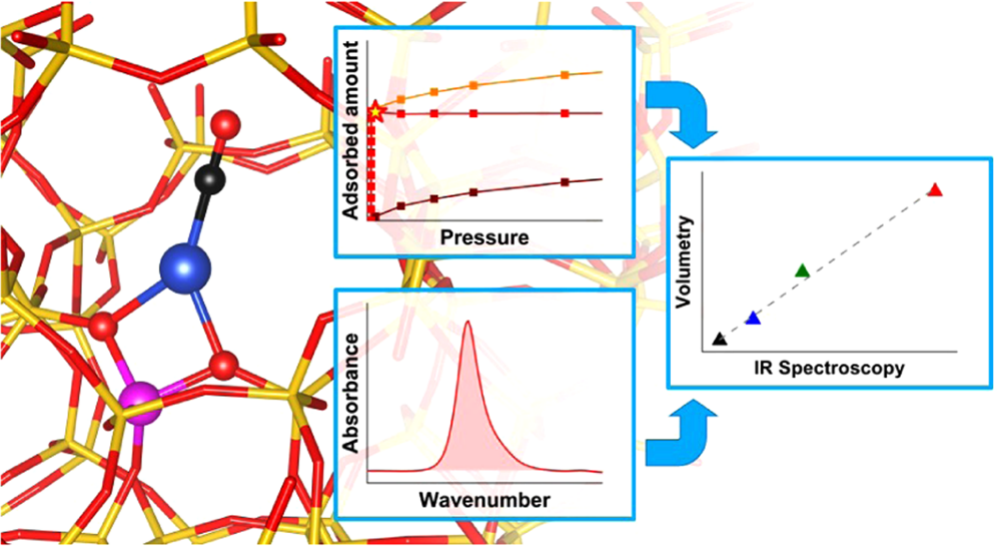

Cu-exchanged zeolites are widely studied materials because of their importance in industrial energetic and environmental processes. Cu redox speciation lies at the center of many of these processes but is experimentally difficult to investigate in a quantitative manner with regular laboratory equipment. This work presents a novel technique for this purpose that exploits the selective adsorption of CO over accessible Cu(I) sites to quantify them. In particular, isothermal volumetric adsorption measurements are performed at 50 °C on a series of opportunely pre-reduced Cu-ZSM-5 to assess the relative fraction of Cu(I); the setup is fairly simple and only requires a regular volumetric adsorption apparatus to perform the actual measurement. Repeatability tests are carried out on the measurement and activation protocols to assess the precision of the technique, and the relative standard deviation (RSD) obtained is less than 5%. Based on the results obtained for these materials, the same CO adsorption protocol is studied for the sample using infrared spectroscopy, and a good correlation is found between the results of the volumetric measurements and the absorbance of the peak assigned to the Cu(I)–CO adducts. A linear model is built for this correlation, and the molar attenuation coefficient is obtained, allowing for spectrophotometric quantification. The good sensitivity of the spectrophotometric approach and the precision and simplicity of the volumetric approach form a complementary set of tools to quantitatively study Cu redox speciation in these materials at the laboratory scale, allowing for a wide range of Cu compositions to be accurately investigated.

## 1 Introduction

Cu-exchanged zeolites have received increasing attention in the last decades for their performance as heterogeneous redox catalysts. The ability of these materials to perform environmentally and industrially crucial reactions, like NH_3_-mediated selective catalytic reduction (SCR) for NO*_x_* abatement^1^ and direct conversion of methane to methanol (DMTM),^2^ led the scientific community to study the mechanisms underlying the peculiar reactivities involved; moreover, the peculiar structural properties of Cu zeolites impart astounding selectivities to these catalysts,^2^ thus encouraging the development of new porous materials also featuring enhanced productivity (e.g., Cu-MOFs).^3−5^ Studying these materials under conditions that are relevant to the reaction they perform is, however, a very difficult task, and many experimental and computational techniques have been exploited in the years to tackle this problem.^6−17^ In particular, the assessment of the oxidation state of the Cu in these materials is of paramount importance, as the Cu(I)/Cu(II) cycle lies at the base of both NH_3_-SCR and DMTM reactions. The techniques that are most frequently applied for this investigation are electronic spectroscopies, such as diffuse reflectance (DR) UV–vis spectroscopy and X-ray absorption spectroscopy (XAS); however, only the latter is applicable for quantitative studies as in the case of UV–vis the molar attenuation coefficient of the species is unknown. Although XAS is currently the gold standard for quantitative Cu redox speciation analysis in these materials,^18^ performing the experiment calls for exploiting large-scale facilities, making it costly, time-consuming, and effectively limiting a thorough understanding of this variable when access to a synchrotron is not feasible. Moreover, quantitative XAS analysis introduces further issues: for example, widely used methods like linear combination fit (LCF) need the spectra of the pure species as input, which are very difficult to obtain for these materials. In this work, the development and validation of a quantitative technique to monitor the amount of Cu(I) based on the selective interaction of CO with Cu(I) in the zeolite framework is presented. By exploiting volumetric isothermal adsorption of CO on a series of Cu-ZSM-5 materials, the amount of accessible Cu(I) after a reductive treatment is assessed with a laboratory adsorption apparatus; furthermore, repeatability tests are carried out to measure the precision of the technique, both in terms of the uncertainty due to the instrumental measurement itself and the one introduced by the pretreatment procedure. The results are used to calculate the molar attenuation coefficient of the IR fingerprint of the Cu(I)-CO adducts, to allow for semiquantitative IR studies in the same context. Other indirect techniques are available for studying Cu(II)/Cu(I) speciation; one of the most frequently used, especially in studies concerning NH_3_-SCR, is temperature-programmed reduction (TPR) with a mixture of NH_3_ and NO.^19,20^ This technique has been successfully applied to titrate the amount of Cu(II) in Cu zeolites, and is thus complementary to the ones proposed herein that aim at titrating Cu(I) sites. When performing NH_3_-NO TPR, the Cu(I) fraction is usually retrieved via mass balance, supposing that no other oxidation states are present other than Cu(I) and Cu(II). In this context, the approach developed in this work has several advantages. First of all, it allows directly measuring the amount of Cu(I), avoiding mass balance in cases where other oxidation states may be present (e.g., Cu(0)); moreover, it can be coupled with NH_3_-NO TPR to retrieve the possible fraction of Cu that is present in states other than Cu(II) and Cu(I) via mass balance. Another advantage of the proposed techniques is the relative ease of use, both in terms of operating conditions (e.g., near-room-temperature measurements) and required instrumentation (adsorption apparatus and/or IR spectrophotometer). Chemisorption of probe molecules and IR spectroscopy have been extensively applied for studying Cu speciation in Cu zeolites,^21−23^ and a recent contribution from Xie et al. applies a complementary experimental and theoretical study to find key indicators for the formation of Cu dimers in Cu-ZSM-5 materials and their relationship with catalytic activity.^24^

The choice of Cu-ZSM-5 materials for this study was based on two main reasons: (i) sharp and narrow IR bands associated with the Cu(I)-CO*_x_* adducts, due to the homogeneity of the environment surrounding Cu species in ZSM-5, (ii) full accessibility of the cations by the probe molecule as all of the substitutional positions for cations are exposed in the channels in the MFI framework. Evidence from IR spectroscopy clearly suggests an irreversible, species-selective chemisorption of CO on Cu(I) sites at 50 °C and at a pressure < 10^–3^ mbar. On the basis of previous reports^9,27^ and as further supported by density functional theory (DFT) investigation reported herein, the stable species are Cu(I) monocarbonyls; thus, the amount of irreversibly adsorbed CO directly titrates the fraction of Cu(I) in the zeolite host. A quantitative approach was developed based on these observations, with the aim of producing an accurate and precise technique for Cu redox speciation studies in these materials.

The same approach could be applied to Cu zeolites belonging to different framework types, obtaining analogous results. However, different topologies are characterized by a less homogeneous environment of Cu species that cause the formation of slightly different Cu-CO*_x_* adducts, implying larger bandwidths and small shifts in the band position.^25−29^ Moreover, in the case of some topologies, Cu location can hamper the accessibility of some Cu species that are consequently hardly probed by CO. Examples are the cations located inside the sodalite cavities in FAU or LTA^30−35^ or in the side pockets of MOR.^36,37^

## 2 Experimental Section

### 2.1 Materials

Two commercial ZSM-5 samples were used for this study: CBV 2314 (Zeolyst International, Si/Al ratio: 11.5, nominal cation form: ammonium, Na_2_O weight %: 0.05, data from producer) and CBV 5524G (Zeolyst International, Si/Al ratio: 25, nominal cation form: ammonium, Na_2_O weight %: 0.05, data from producer). Initially, the NH_4_-ZSM-5 samples were exchanged by a 1 M NH_4_NO_3_ solution at 70 °C for 6 h, to obtain the sample in a purely ammonium form. The exchange procedure was repeated three times, supplying fresh NH_4_NO_3_ solution at each stage. At the end of the third repetition, the sample was washed with abundant distilled water and dried at 70 °C for 2 h. Finally, the amount of Cu(II) acetate monohydrate (Sigma-Aldrich, 99.99%) required to obtain a concentration of 1 and 20 mM (for lower and higher Cu loadings, respectively) was dissolved in water and the ammonium form of the zeolite was added to the solution (250 mL per zeolite gram). The mixture was stirred at room temperature for 48 h and then the obtained Cu zeolite was filtered and washed. The procedure was repeated twice for selected samples, to approach stoichiometric exchange (Cu/Al = 0.5). Finally, the exchanged zeolite as recovered by filtration was dried at 100 °C overnight and calcined at 550 °C for 5 h to remove the residual acetate. In all cases, the solution was buffered with acetic acid in the 4–5 pH range, to avoid the formation of extraframework CuO*_x_* species. The Cu loading of the materials, expressed as Cu/Al ratios, was evaluated by inductively coupled plasma optical emission spectroscopy (ICP-OES): the composition of the obtained materials is summarized in Table 1.

**Table 1:** List of Cu Zeolites Used in This Work^a^

name	Si/Al^a^	Cu/Al^b^
(0.07)Cu-MFI(11.5)	11.5	0.07
(0.35)Cu-MFI(11.5)	11.5	0.35
(0.45)Cu-MFI(11.5)	11.5	0.45^c^
(0.05)Cu-MFI(25)	25	0.05
(0.48)Cu-MFI(25)	25	0.48^c^

a (a) Si/Al ratio supplied by the producer (Zeolyst International). (b) Cu/Al ratios measured by ICP-OES. All Si/Al and Cu/Al are reported as molar ratios. (c) Exchange procedure repeated twice.

### 2.2 Methods

Carbon monoxide (supplied by Sapio SRL, 99.99998% purity grade) adsorption isotherms were measured on a commercial volumetric apparatus (Micromeritics ASAP 2020, Norcross GA) at 50 °C. This specific temperature was selected since it is estimated for a sample exposed to an IR beam during an IR experiment, where precise Cu-carbonyl stability is expected (vide infra). The samples were ground in a mortar and pelletized, to prevent any powder residues from moving out of the cell while exposed to gas/vacuum treatment. The pellets were then inserted in a custom adsorption cell (Figure S1) consisting of a quartz burette with connections that allow for vacuum and thermochemical treatment; the cell is equipped with a plug-in thermal jacket for measurements in thermostatic fluid.^38^ The sample temperature was kept constant using an external isothermal liquid bath (Julabo F25-EH). Prior to the measurements, Cu zeolite pellets were treated at a high temperature on a vacuum line equipped with a turbomolecular pump; full details on the treatment can be found in the next section. The sample is usually measured in the form of pellet fragments, to avoid possible damage to the instrumentation; depending on the amount of sample and cell volume, the finer part of a powder could escape the cell if the pressure is abruptly changed (e.g., if evacuating starting from a high pressure). If the measurement strictly needs to be performed on a powder (e.g., if there is proof that the form of the sample may influence the composition, or if it is directly sampled out of a catalytic bed), the instrumental parameters can be tuned to perform safe experiments; for instance, the outgassing can be performed with smaller, more frequent steps.

Transmission IR spectra were acquired using an Invenio R spectrophotometer from Bruker, equipped with a mercury cadmium telluride (MCT) cryodetector, a resolution of 2 cm^–1^ and averaging 32 scans (64 for background spectrum, collected with an empty measurement chamber). The samples were sieved and pressed into self-supporting pellets, placed in a gold envelope, and inserted in a quartz cell equipped with KBr windows (optical path: 2 mm); to be able to calculate the molar attenuation coefficients, the area and the weight of the pellets were measured. For transmission IR spectrophotometry, the sample needs to be pressed into a pellet form to guarantee uniform thickness and to minimize scattering effects; this implies that samples in powder form cannot be measured as such by employing this technique.

### 2.3 Activation Protocol

For the coupled IR/volumetry experiments on all samples, the activation protocol was performed as follows. The sample cell was heated from room temperature to 550 °C with a ramp of 5 °C/min in dynamical vacuum. O_2_ (100 mbar) was then dosed and kept in contact with the sample at the same temperature for 30 min. The sample was then outgassed at the same temperature for 40 min. Under these conditions, most of the Cu in the samples was converted to Cu(II), as suggested by the almost absent (within the detection limit of conventional XANES) 1s > 4p transition due to Cu(I) (8983 eV) in the XAS spectrum of the material (see Figure S2). Then, a controlled NH_3_ treatment was performed to maximize the amount of Cu(I). Previous data showed that prolonged treatment in NH_3_ at a high temperature (i.e., 500 °C) can reduce 75–90% of the total Cu to Cu(I).^39^ This result is qualitatively supported by XANES data collected for the (0.45)Cu-MFI(11.5) sample (Figure S2). Accordingly, 100 mbar of NH_3_ was dosed on the sample at 550 °C for 30 min. Finally, outgassing was performed at the same temperature until a residual pressure of 5 × 10^–4^ mbar was achieved, then the sample was cooled down to room temperature. This procedure aimed to maximize the amount of highly uncoordinated Cu(I) that would have been probed by CO.

For both repeatability tests, the activation protocol was performed as follows. The sample was heated from room temperature to 500 °C with a ramp of 5 °C/min in dynamical vacuum. O_2_ was then dosed and kept in contact with the sample at the same temperature for 1 h. The sample was then outgassed at the same temperature for 40 min. NH_3_ was dosed on the sample at 500 °C for 1 h. Finally, outgassing was performed at the same temperature for 1 h and the sample was cooled down to room temperature. The only difference between the two activation protocols was the partial pressure of the gases (300 vs 100 mbar) to take into account the different amounts of sample used (ca. 300 vs 80 mg per replica).

### 2.4 Computational Details

The (co-)adsorption of CO and NH_3_ on Cu-ZSM-5 was simulated at the DFT level of theory by means of the CRYSTAL17 periodic code.^40^ ZSM-5 models and computational parameters were adapted from the previous report by Morra et al.^41^ In brief, Cu(I) substitution was investigated at three different sites, i.e., those with Al occupying the T7, T8, and T10 tetrahedral positions in the MFI framework. The Al siting has been chosen on the basis of occupancies experimentally obtained by X-ray diffraction (XRD) on Cs-ZSM-5 samples.^42^ The calculations were carried out with the hybrid GGA B3LYP functional.^43,44^ Dispersive interaction has been included empirically through the Grimme D3 scheme.^45^ Concerning the basis set, the framework atoms have been described through a double-ζ quality basis set; in detail, the basis set proposed by Nada and co-workers was adopted for Si and O atoms,^46^ whereas the Al was described with the basis set from Catti et al.:^47^ such choice provides a good description of zeolitic frameworks at a reasonable computational cost.^41,48−50^ Concerning the extraframework Cu cations, as well as atoms of sorbed molecules, these have been described through the Ahlrichs TZVP basis.^51^ The truncations for the mono- and bielectronic integral (TOLINTEG) were set to {7 7 7 7 25}. The sampling in the reciprocal space (SHRINK) was set to {2 2}, for a total of 8 k points. The maximum order of shell multipoles in the long-range zone for the electron–electron Coulomb interaction (POLEORDR keyword) was chosen to be 6. All of the other parameters were set to default values according to the CRYSTAL17 manual.^52^

Each Cu(I) model was geometry optimized and, upon screening, T10 was revealed to be significantly more stable than T7/T8, accounting for 99% of the substituted sites on a Boltzmann population basis. Accordingly, the adsorption processes were simulated only for the T10 site. Molecular adducts were manually built and further optimized. The following adducts were considered: Cu(I)(CO), Cu(I)(CO)_2_, Cu(I)(NH_3_), and Cu(I)(NH_3_)(CO). The main geometrical parameters for each structure are given in Table S1. To evaluate the vibrational properties and the thermodynamic functions describing the Cu adducts (enthalpies and Gibbs free energies), harmonic frequencies were computed for a subset of atoms including the Cu(I) cation; the sorbed molecule(s); and the Al framework atom and its neighbors up to the second coordination shell (namely, 4 O and 4 Si atoms).

The variation of electronic energy (Δ*E*), enthalpy (Δ*H*), and Gibbs free energy (Δ*G*) associated with the formation of an adduct, evaluated under experimentally relevant *p*, *T* conditions, were computed as follows
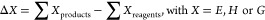


The relevant processes for which formation/dissociation energetics have been computed, selected on the basis of experimental evidence, are summarized in Table 2.

**Table 2:** Relevant Processes of Cu(I) Adduct Formation/Dissociation Investigated by DFT

#	process	
1	Cu(I)(CO) formation	ZCu(I) + CO → ZCu(I)(CO)
2	Cu(I)(CO)_2_ formation	ZCu(I)(CO) + CO → ZCu(I)(CO)_2_
3	Cu(I)(NH_3_) formation	ZCu(I) + NH_3_ → ZCu(I)(NH_3_)
4	Cu(I)(CO)(NH_3_) formation	ZCu(I)(NH_3_) + CO → ZCu(I)(CO)(NH_3_)

## 3 Results and Discussion

### 3.1 CO Adsorption on Cu Zeolites

**Figure 1:**
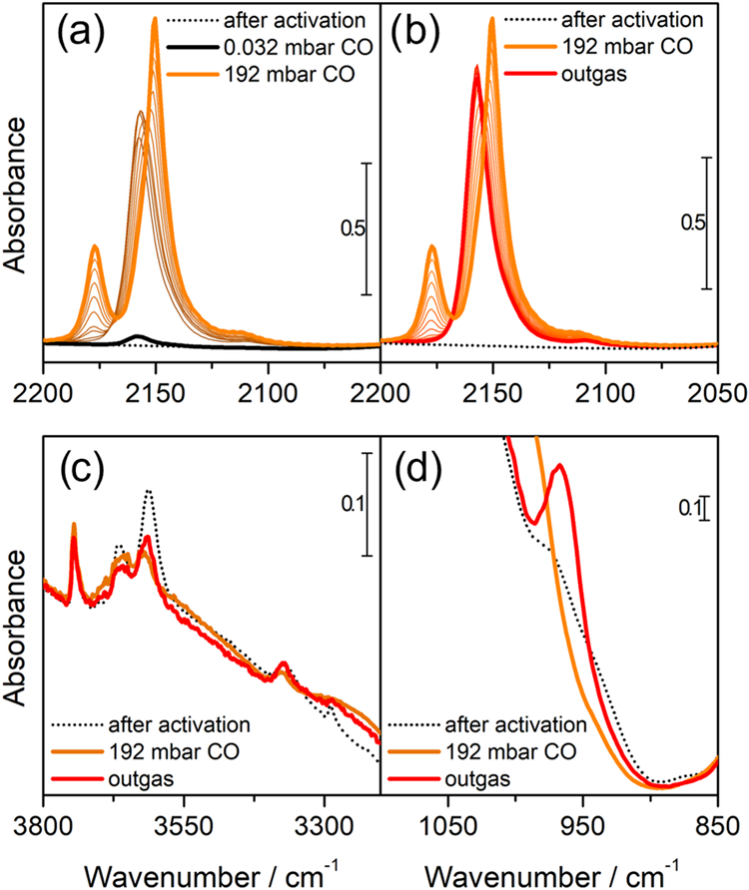
Interaction of CO over the pre-reduced (0.35)Cu-MFI(11.5) sample. (a) Effect of increasing pressure of CO (from black to orange). (b) Effect of outgassing on the sample exposed to 192 mbar of CO (from orange to red). (c) Effect of CO on the OH stretching modes. (d) Effect of CO on the perturbation of framework modes by Cu.

Detailed characterization of the interaction of CO with Cu-exchanged zeolites has been available for more than 20 years.^9,27,53,54^ In particular, IR and X-ray spectroscopies highlight how the interaction of CO with Cu(I) sites is heavily dependent on the temperature and the partial pressure of CO, while interaction with Cu(II) is generally negligible at ambient conditions. At 50 °C, and generally near room temperature, increasing pressures of CO favor the formation of Cu(I)CO and Cu(I)(CO)_2_ adducts in a subsequent manner, as is evident from IR spectra of CO dosed on a pre-reduced material (Figure 1). For Cu-ZSM-5 materials, as already reported in the literature, an absorption band is first formed at 2157 cm^–1^ corresponding to the CO stretching frequency in the Cu-monocarbonyl adducts; when pressure is increased, this band starts decreasing in intensity in correspondence with the appearance of two bands at 2178 and 2151 cm^–1^, assigned to the symmetric and antisymmetric stretching of the CO molecules in Cu-dicarbonyl adducts (along with the rotovibrational profile of the CO in the gaseous phase) (Figure 1a).^54^ An additional weak band can be noticed at 2108 cm^–1^ when the CO is first dosed and when the sample is outgassed: this has been assigned to the stretching mode of Cu(I)^13^CO adducts, based on the relative intensity and position of this band (matching the expected isotopic shift, as estimated via harmonic oscillator model, of −48 cm^–1^) with respect to the Cu(I)^12^CO one.^55−58^ The spectra are very well defined with bands characterized by a small bandwidth, very similar to what would be expected in the case of a homogeneous complex. This suggests the formation of uniform complexes characterized by a very similar structure.^53^ If the sample is evacuated, the trend is reversed and the single band corresponding to the Cu-monocarbonyls is restored (Figure 1b); thanks to the depletion of Cu dicarbonyls, the intensity of the monocarbonyls band reaches its maximum intensity. Further evacuation does not change the band intensity even at long time scales (several hours), suggesting an irreversible interaction between the Cu(I) and the adsorbed CO.

The high stability of the Cu(I)CO, together with its unusually high CO stretching frequencies have been explained in terms of Cu(I)CO bonds where electrostatic and σ-dative covalent contributions are predominant, as well as contribution from π-back-donation.^29^

After activation, the material presents three main peaks in the 3800–3500 cm^–1^ region (Figure 1c), assigned, respectively, to the O–H stretching modes of isolated silanols on the surface (3745 cm^–1^), partial extraframework AlOH species (3664 cm^–1^), and Al(OH)Si Brønsted sites (3610 cm^–1^).^59,60^ Upon CO dosages at room temperature, we do not expect any erosion of the Brønsted sites, due to the low proton affinity of the probe;^61^ conversely, as soon as CO is introduced, both signals associated with the stronger acidic protons (i.e., AlOH and Al(OH)Si) are partially consumed. This unexpected fact can be explained considering the presence of traces of NH_3_ coordinated to Cu sites, that are displaced once CO is admitted in the cell and can further react with the acidic proton sites and form some NH_4_^+^ species. The formation of NH_4_^+^ explains the decrease in intensity of the band peaked at ca. 3300 cm^–1^ (assigned to a N–H stretching of residual NH_3_), as well as the growth of broad bands centered at 2963, 2800, and 2598 cm^–1^ and the peak at 1450 cm^–1^, due to the ammonium bending mode^62^ (Figure S3). A direct interaction of CO with the Brønsted acid sites is thus excluded, based on the absence of any characteristic peak associated with this adduct (1169–1175 cm^–1^); furthermore, the literature agrees that this interaction is only present at significantly lower temperatures (e.g., liquid N_2_ temperature).^63^ The displacement of NH_3_ from Cu sites appears quantitative upon CO dosage; thus, the residual fraction of coordinated ammonia is not affecting the Cu(I) titration by CO.

This vision is further confirmed by inspecting the 1050–850 cm^–1^ zone of the IR spectra (Figure 1d): for Cu-exchanged zeolites in which the cation can interact strongly with the framework (i.e., it is barely screened by ligands) the appearance of one or more peaks associated to the cation-perturbed framework modes is expected.^53^ As can be seen, only a small absorption at 979 cm^–1^ is present after activation; when a high pressure of CO is introduced, this shoulder disappears, and a stronger and sharper contribution appears at 967 cm^–1^ after the sample is outgassed. This is consistent with an initial coordination of Cu by NH_3_ molecules that are readily displaced by CO, which coordinates the Cu sites upon increasing pressure as Cu dicarbonyls; when the sample is outgassed, the Cu(I) reverts to stable monocarbonyl complexes without further coordination by NH_3_ (suggested by the appearance of the strong peak at 967 cm^–1^ and the fact that the Brønsted protons in the high-frequency zone do not recover their initial intensity).

A similar trend was observed in all of the samples, apart from the appearance of an additional component at 2133 cm^–1^ when CO pressure is increased, mainly visible in the case of the (0.48)Cu-MFI(25) material. This peak has been assigned to a mixed ligand [Cu(NH_3_)(CO)]^+^ complex,^53^ testifying that for this sample, the removal of NH_3_ was less effective (Figures S4 and S5). However, the presence of some NH_3_ does not invalidate the measurement with CO, due to the stronger interaction of CO that displace NH_3_. For the sake of completeness, the data obtained on this sample are reported in the supporting.

### 3.2 Description of Volumetric Methodology and Validation

**Figure 2:**
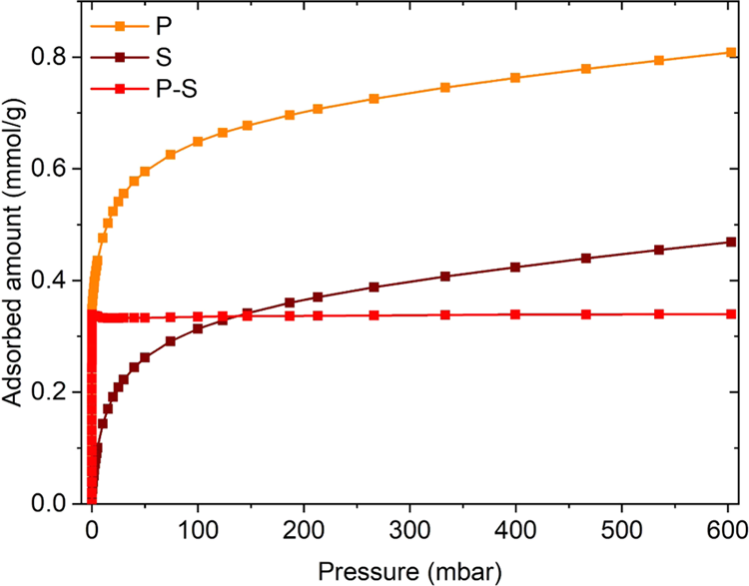
CO adsorption isotherms performed at 50 °C on the pre-reduced (0.35)Cu-MFI(11.5) sample. Orange: primary isotherm (P); brown: secondary isotherm (S); red: difference between the primary and secondary isotherm (P–S), used to calculate the amount of irreversibly bound CO.

Based on the phenomenon described above, isothermal volumetric adsorption measurements of CO at 50 °C (i.e., the temperature of the sample under the IR beam) can be exploited to quantify the amount of Cu(I) accessible by this gaseous probe. The procedure works as follows. A primary CO adsorption isotherm is collected for the treated sample: when the gas is dosed, the probe is adsorbed on the Cu sites with a species distribution dictated by the pressure (as detected by IR spectroscopy). When a plateau is reached, most of the CO is adsorbed on Cu(I) sites as dicarbonyls; upon evacuation, these revert to Cu(I) monocarbonyls; thus, only the fraction of irreversibly adsorbed CO is retained. Subsequently, a secondary CO adsorption isotherm is collected; since the Cu(I) sites are already bonding a CO molecule each, the secondary isotherm only accounts for the reversible CO fraction (i.e., Cu dicarbonyls). By subtracting the two isotherms, the value at the elbow point represents the amount of CO irreversibly bound to the catalyst (in mmol/g); since the composition of the catalyst is known and the irreversibly bound CO is in a 1:1 ratio with the Cu(I) sites, the percentage of Cu(I) over the total amount of Cu can be easily calculated. An example of this measurement for a Cu-ZSM-5 sample is shown in Figure 2. The complete dataset of isotherms for all considered materials is reported in Figure S6.

The concentration of reduced Cu detected by the volumetric technique is in line with XAS results (see Figure S2), so repeatability tests were performed to assess the precision of this method for quantitative purposes; the (0.35)Cu-MFI(11.5) material was used for all of these tests. Two main sources of uncertainty for this technique were identified, being the uncertainty intrinsic to the volumetric measurement itself and the one associated with the treatment procedure, and two separate sets of experiments were devised to calculate them.

Since errors associated with the pretreatment were estimated to be higher than the ones related to the measurement itself, all possible interferences from the activation procedure were excluded for the first step. This was done by activating a large batch of the sample and storing it in an Ar-filled glovebox (residual O_2_ and H_2_O concentrations < 0.5 ppm); an adequate portion of this identical sample was taken time after time for three replica measurements, which were compared to check instrumental repeatability. The results are summarized in Table 3.

**Table 3:** Results of the Instrumental Repeatability Tests on the (0.35)Cu-MFI(11.5) Sample

replica	irreversibly adsorbed CO (mmol/g)	Cu(I) concentration (%)
1	0.3389	75.2
2	0.3231	71.7
3	0.3110	69.0

As can be noticed, the amount of adsorbed CO shows a decreasing trend in time, which is reflected in the Cu(I) concentration. Each measurement can take up to 24 h, so this decrease could be ascribed to small amounts of pollutants inside the glovebox. For instance, very small amounts of water inside the inert environment could potentially lead to Cu(I) oxidation to Cu(II) over long periods of time, as has been already reported in the literature.^64^ It has been also shown that introducing H_2_O in such materials when CO is interacting with Cu(I) (i.e., when monocarbonyls are present) can lead to mixed ligand H_2_O/CO adduct,^53^ which are, however, reversible in vacuum and do not seem to be able to oxidize Cu(I). Regardless of the source of the pollution, it has to be stressed that under normal operating conditions (i.e., not in conditions similar to this test, in which a sensitive sample has been stocked for a long time because of the way the experiment had to be performed), the sample is measured in the same cell in which it is activated, and so possible pollutants are unlikely to reach the material during transfer from the vacuum line to the instrument. It should also be noted that the standard measurement procedure does not include any transfer in the glovebox, so this particular interference is not an issue for the actual measurements. The calculated RSD of the results from this test is 4.3%, although if a systematic error is present in this case due to sample pollution over time, this may not be an accurate indicator for measurement uncertainty. Nonetheless, the sources of uncertainty strictly related to the measurement procedure should include instrumental parameters that are set or measured by the instrument. In particular, supposing repeated measurements on samples that have the same exact composition (in terms of Cu(I) amount), errors can arise from weighing the sample in the cell (as well as the empty cell before the sample is introduced), dosing and measuring the gas pressure (done automatically by the volumetric instrument), equilibration times, small drifts in the isothermal bath and other similar effects. Overall, each of these sources of uncertainty is reported and certified for the instruments (balances, volumetric apparatus, thermocouples, etc.) and can, in principle, be used to calculate a total measurement-related uncertainty through the propagation of error. The actual value for this kind of uncertainty will depend on the specific instruments adopted, and it is outside the scope of this work to extensively discuss these aspects.

The uncertainty associated with the treatment was subsequently calculated; the procedure involved the activation and measurements of three separate replicas of the same sample that were prepared and measured one after the other in separate cells. The results are reported in Table 4.

**Table 4:** Results of the Treatment Repeatability Tests on the (0.35)Cu-MFI(11.5) Sample

replica	irreversibly adsorbed CO (mmol/g)	Cu(I) concentration (%)
1	0.4123	91.5
2	0.4115	91.4
3	0.3801	84.4

As expected, the uncertainty related to the treatment is slightly higher (relative standard deviation: 4.58%); this can be ascribed to small differences in the activation procedure such as gas pressures not being exactly the same, slight differences in the temperature and exposition time, different times between disconnecting the cell from the vacuum lines and connecting it to the volumetric apparatus, and so on. Overall, though the error is not very high, it is advisable to operate in replicas when possible. The higher Cu(I) content detected by this experiment compared to the previous one can be ascribed to different effects, namely, (i) the overall simpler and shorter procedure, (ii) the smaller volume of the cell, and (iii) the lower amount of pelletized sample. It should be noted, however, that both of these experiments aimed at assessing the repeatability of their respective step of the procedure, i.e., calculating the variance of the different replicas; differences in the mean value for the two experiments are thus expected, as the procedure for the two experiments is different.

### 3.3 Insights from Periodic DFT Calculations

Table 5 lists the energetic values obtained for the formation of simulated adducts from DFT calculations.

**Table 5:** Δ*E*, Δ*H*, and Δ*G* Values (in kJ/mol) Computed for the Formation of Cu(I)-CO/NH_3_ Adducts Listed in Table 2 (*T* = 50 °C)

		Δ*H*		Δ*G*	
adduct #	Δ*E*	*p* = 0.001 mbar	*p* = 100 mbar	*p* = 0.001 mbar	*p* = 100 mbar
**1**	–169.3	–163.1	–163.1	–80.3	–111.2
**2**	–65.8	–72.5	–63.6	12.2	–9.9
**3**	–203.2	–192.9	–192.9	–107.6	–138.5
**4**	–81.3	–78.8	–78.8	6.6	–24.3

All of the adsorption events simulated here do not cause any significant deformation of the MFI framework, as testified by the negligible modification of the cell parameters and, consequently, of the cell volume; the local surrounding of Cu(I) is, instead, significantly affected (see Figures S7, S8 and Table S1). The subsequent adsorption of two CO molecules brings the Cu(I) from its original bipodal coordination to the framework O atoms toward a quasi-tetrahedral coordination environment, passing through a planar, triligated structure upon the adsorption of a single CO molecule.^65^ The adsorption of a NH_3_ molecule induces an even more severe deformation, with Cu(I) coordinated in an almost linear geometry between a single-framework O and the adsorbed molecule, as already proposed for other Cu-CHA during NH_3_-temperature programmed desorption (TPD) experiments.^17^ By adsorbing a further CO molecule, the coordination turns back to a more regular quasi-tetrahedral environment. Thermodynamic functions were evaluated at conditions relevant for the sake of experiments interpretation, i.e., at a temperature of 50 °C and at values of pressure representative for a complete adsorption (100 mbar) and for a full desorption of reversible fractions (0.001 mbar). Overall, all of the simulated processes are exothermic, with a large dependence of the evolved heat on the number of coordinated molecules (i.e., significantly lower in the event of the adsorption of a second molecule); the computed values are in good agreement with calorimetric data previously reported in the literature.^66^ Furthermore, the variation of the pressure has a limited effect on Δ*H* values. Instead, this impacts much more in the Δ*G* values: as a matter of fact, most of the processes are exoergonic, but some turn to endoergonic at the lower pressure value. In detail, the monocarbonyl adduct (**1**) is always stable, regardless of the considered pressure. Conversely, the dicarbonyl adduct (**2**) is stable only at high pressures, whereas it is expected to easily desorb as the pressure is decreased, in line with experimental observations. The same behavior commented for **1** is also observed for the ammonia adduct (**3**) that exhibits the highest stability among the considered structures. Finally, the formation of a mixed NH_3_-CO adduct (**4**), where a CO molecule is adsorbed on a preexisting ammonia adduct (**3**), is favored at a high pressure, whereas it turns slightly unstable upon pressure decrease. The overall description by DFT fully supports the experimental observations: the superior stability of monocarbonyls against dicarbonyls enforces the assumptions at the basis of the volumetric titration method. Also, the polluting effect of residual ammonia is highlighted, as in the case of isolated Cu(I) sites: (i) the NH_3_ adduct is more stable than the CO one, inferring the CO adsorption event could not in principle displace a preadsorbed ammonia molecule; and (ii) the stability of the mixed adduct is not achieved at a low pressure. Thereby, Cu(I) sites interacting with residuals of NH_3_, not desorbed during the material activation, cannot be titrated by CO within the proposed experimental conditions, thus constituting a relevant contribution to the treatment-related uncertainty. However, the residual amount of NH_3_ is small (as from IR measurements); thus, the untitrated Cu(I) fraction is most probably contributing to the error of the method. Furthermore, IR spectroscopy experimentally shows that part of the NH_3_ is displaced to the Brønsted acid sites through a mechanism not described by our simplified DFT model. The contribution of NH_3_ pollution is then even lower than expected from the bare simulation results.

### 3.4 Calculation of Molar Attenuation Coefficient

**Figure 3:**
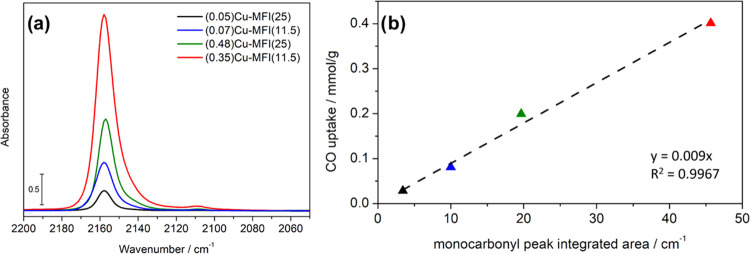
CO adsorbed on the four reference Cu-ZSM-5 samples. (a) IR spectra of the samples after interaction with 200 mbar CO and outgassing (spectra have been normalized, and the spectrum of material prior to CO dosage has been subtracted). (b) Uptake of irreversibly bound CO on the samples versus integrated area of the Cu(I) monocarbonyl species. The intercept has been fixed to 0.

Once the precision of the volumetric methodology was assessed, the same protocol was used to measure the amount of accessible Cu(I) in all Cu-ZSM-5 samples. All of the materials show a total Cu(I) content of around 85% after NH_3_ reduction at 550 °C and are in very good correlation with the IR data collected on samples treated under the same conditions and exposed to CO (Figure 3).

**Figure 4:**
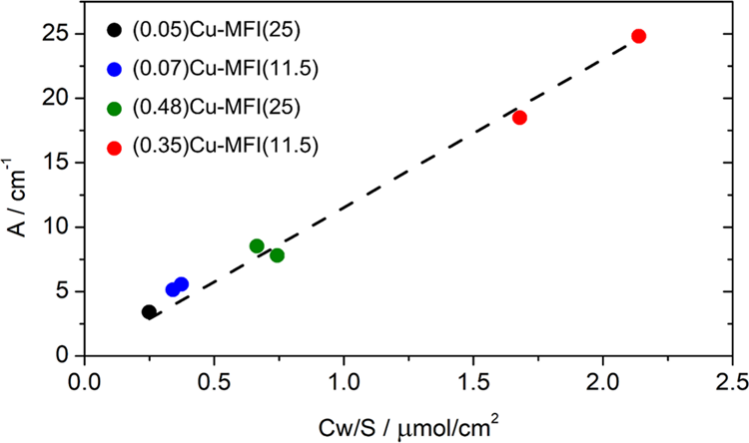
Linear model for quantifying the molar attenuation coefficient relative to the Cu(I) monocarbonyl adducts. For the (0.07)Cu-MFI(11.5), (0.35)Cu-MFI(11.5), and (0.48)Cu-MFI(25) samples, two replicas were measured. The intercept has been fixed to 0.

The spectra reported in Figure 3a show the interaction of CO with the reduced samples, and have been treated to obtain comparable data; full information on the normalization procedure can be found in the SI (Section S6). As can be seen in Figure 3b, the correlation with the volumetric data acquired on the four samples is very good. This agreement suggests that IR spectrophotometry can be used as a semiquantitative tool for the same purpose if the molar attenuation coefficient of the Cu(I) monocarbonyl vibration is known. The application of the Beer–Lambert law to solid materials is not trivial, and one should be aware that scattering effects and differences between the geometrical and the optical thickness can lead to incorrect results.^67^ These conditions must be checked *a posteriori* (see Section S7, Figures S9 and S10 in the SI for the complete statistical analysis), and the molar attenuation coefficient can be obtained by applying the following equation:
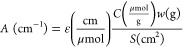
where *A* is the integrated area of the band of interest, ε represents the integrated molar attenuation coefficient of the band, *C* is the concentration of the species (in this case, the concentrations resulting from the volumetric technique were used), and *S* and *w* are the geometrical area and the weight of the sample pellet, respectively. By plotting *A* vs *Cw*/*S*, the molar attenuation coefficient of the band is the slope of the line, as shown for this case in Figure 4.

To retain the quantitative aspect of this analysis, the integrated areas of the monocarbonyl peaks are calculated on the non-normalized spectra after subtraction of the spectra of the corresponding activated material. The model is linear in a range corresponding to a large spectrum of possible compositions of Cu-ZSM-5 materials and Cu zeolites in general (0.16–2.6 Cu wt %), and the molar attenuation coefficient obtained with this method corresponds to 11.5 ± 0.3 cm/μmol; details on the statistical analysis can be found in the SI. In 2012, Góra-Marek et al.^25^ performed a similar analysis on one sample of Cu-ZSM-5 and used the results to determine Cu speciation in Cu zeolites of different topologies. In their study, they decided to use the absorbance instead of the integral of the peak, so the two molar attenuation coefficients cannot be directly compared, also because of the likely difference in pellet weight. By fitting the data obtained in the present work using a model that employs absorbance instead of the integral of the peak, a similar (not integral) molar attenuation coefficient is obtained (1.2 instead of 1.3 cm^2^/μmol); this discrepancy is most likely due to a different sample set (i.e., a wider range of Cu(I) concentrations) and the decision not to include the origin point (0,0) explicitly in the dataset. As a matter of fact, using the equation proposed in the present work allows comparison between materials with different compositions and, most importantly, bandwidth is somehow taken into account by the use of the integrated molar attenuation coefficient. In particular, the same interaction on zeolites that bear different topologies may show different bandwidths and band symmetries due to the relative inhomogeneity of the sites; this, in turn, may lead to inaccurate quantitative results if the absorbance value is used instead of the peak integral. Finally, it is important to note how the value for the integrated molar attenuation coefficient is rather high; as a comparison, the integrated molar attenuation coefficient for pyridine adsorbed on Brønsted acid sites on zeolites is ε(B)_1545_ = 1.02 cm/μmol. This confirms the high sensitivity of the technique, making its application advantageous even when the Cu(I) content is low (low Cu content of the sample and/or low reduced fraction). For very high Cu(I) contents, the absorbance may exceed the limit in which the Beer–Lambert law can be safely applied; the volumetric approach is, however, an accurate technique even for these cases. In this sense, the two techniques can be thought of as complementary since volumetric measurements may not be as accurate for samples that show too low adsorption.

## Conclusions

The selective interaction of CO with Cu(I) has been exploited to develop a novel technique to quantitatively assess the amount of accessible Cu(I) sites in Cu zeolites, using a set of Cu-ZSM-5 with variable composition as reference materials. This volumetric adsorption technique allows us to access Cu redox speciation, a key variable in many catalytic processes based on this class of materials, by means of common laboratory instrumentation; in addition, repeatability tests on the measurements further reported a good precision for this methodology (relative error < 5%). This quantification, applied to a selected range of Cu-ZSM-5 materials, nicely correlates with the area of the IR band associated with Cu(I) monocarbonyl species; by modeling this correlation with the Beer–Lambert law, the molar attenuation coefficient for this peak has been calculated, allowing for easily accessible semiquantitative IR studies on these materials. Future work will expand this concept toward different zeolites, to explore the applicability of these methods to materials with more complex topology (e.g., featuring potentially inaccessible Cu(I) sites) but more relevant for their catalytic activity.

## Supporting Information Available

Volumetric apparatus; XANES spectra of oxidized and reduced (0.45)Cu-MFI(11.5) material; mixed ligand NH3/CO complex from IR spectroscopy; CO adsorption isotherms; DFT optimized structures; normalization of the IR spectra of the materials interacting with CO; and statistical analysis of the spectrochemical linear model (PDF)
