# Supplementary material for: Titration of Cu(I) Sites in Cu-ZSM-5 by Volumetric CO Adsorption
Source: ACS Appl Mater Interfaces. 2022 Apr 28;14(18):21059–68. doi: 10.1021/acsami.2c03370 (PMC9100488; doi:10.1021/acsami.2c03370)
Supplement: Supplementary file 1 — am2c03370_si_001.pdf [file am2c03370_si_001.pdf]

Supporting Information for

Titration of Cu(I) sites in Cu-ZSM-5 by volumetric CO adsorption

Gabriele Deplano[1], Matteo Signorile[1*], Valentina Crocellà[1], Natale Gabriele Porcaro[1], Cesare Atzori[1§], Bjørn Gading Solemsli[2], Stian Svelle[2] and Silvia Bordiga[1*]

[1]Department of Chemistry, NIS and INSTM Reference Centre, Università di Torino, Via P. Giuria 7 - 10125 and Via G. Quarello 15/A -10135, Torino (TO), Italy

[2]SMN Centre for Materials Science and Nanotechnology, Department of Chemistry, University of Oslo, P.O.Box 1033 Blindern, NO N-0315 Oslo, Norway

[§] Present Address: C.A. European Synchrotron Radiation Facility, 71 Av. des Martyrs, 38000 Grenoble, France

** Corresponding authors: Dr. Matteo Signorile, matteo.signorile@unito.it; prof. Silvia Bordiga, silvia.bordiga@unito.it*

Index

1. Details on the volumetric apparatus S2
2. XANES spectra of oxidized and reduced (0.45)Cu-MFI(11.5) material S3
3. Details on the mixed ligand NH3/CO complex from IR spectroscopy S4
4. CO adsorption isotherms S7
5. DFT optimized structures S8
6. Normalization of the IR spectra of the materials interacting with CO S9
7. Details on the statistical analysis for the spectrochemical linear model S10
References S11

1. Details on the volumetric apparatus

The cell that has been employed for volumetric measurements is shown in ***Figure S1***. It is composed of a quartz burette that contains the sample, linked to a valve that can be connected to a vacuum line enabling thermochemical treatment and exposure to different reactants in the gas phase. The sample environment can be inserted in a thermal jacket that can be filled with a thermostatic fluid, allowing for precise isothermal conditions.

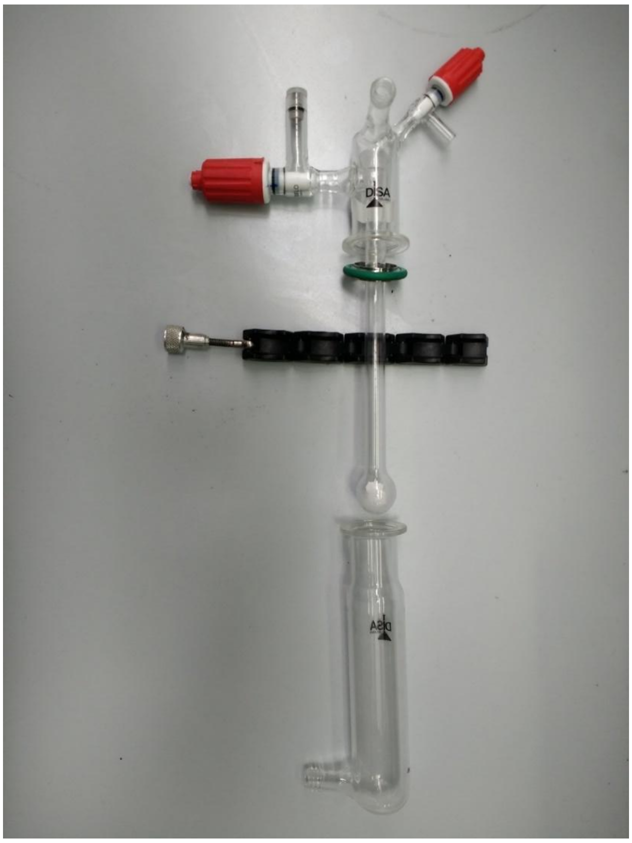

Figure S1. Picture of the cell employed for isothermal volumetric adsorption measurements. Italian patent application n°102020000005014 filed on March 9th, 2020 and PCT n. PCT/IB2021/051769 filed on March 3rd, 2021.

2. XANES spectra of oxidized and reduced (0.45)Cu-MFI(11.5) material

Cu K-edge XAS spectra were collected on the (0.45)Cu-MFI(11.5) material, selected as representative sample, at the BM31 beamline at ESRF (Grenoble), in transmission mode using a Si(111) double-crystal monochromator. The incident (I_0) and transmitted (I_1) X-ray intensities were detected in the range within 8780 and 10500 eV (40 min/scan, 3 spectra collected per sample) using two ionization chambers filled with a mixture of He and Ar. Finally, a third ionization chamber (I_2) was used for the simultaneous collection of a Cu metal foil XANES spectrum employed in the energy calibration procedure. The collected XAS spectra

were then aligned in energy and normalized to unity edge jump using the Athena software from the Demeter package[1]. The samples were prepared for XAS measurements in the form of self-supporting pellets, with thickness optimized for transmission measurements. The pellets were treated as for the IR/volumetric measurement, also interrupting the treatment after the pre-oxidation of the material in order to collect a reference spectrum of a deeply oxidized state. After treatment, pellets have been transferred in a N_2-filled glovebox (O_2 and H_2O concentrations < 0.5 ppm) and sealed in polyethylene bags in order to avoid the re-exposure to the external environment. The measurements have been performed with the pellets still inside the plastic envelope, guaranteeing the desired chemical state is maintained. ***Figure S2*** depicts the XANES spectra of the (0.45)Cu-MFI(11.5) material after exposure to O_2 and NH_3 at 500°C. As can be seen from the spectral profiles, the two states are characteristic of deeply oxidized (Cu(II)) and reduced (Cu(I)) states respectively, as already reported in the literature for these materials[2]. The absence of an evident 1s→3d transition (8977.5 eV) in the spectrum of the material reduced in NH_3 at 500 °C suggests the residual fraction of Cu(II) is certainly lower than 10%. The sharp 1s→4p transition (8983 eV) is instead confirming the existence of low-coordinated Cu(I) sites, compatibly with a prevalence of “naked” Cu(I) ions directly interacting with the zeolite framework. In the case of a major contribution of (quasi-)linear $Cu(I)(NH_3)_x$ (with x = 1 or 2) adducts, a higher intensity for the 1s→4p transition, i.e. comparable with that of the white line peak, would have been expected[3].

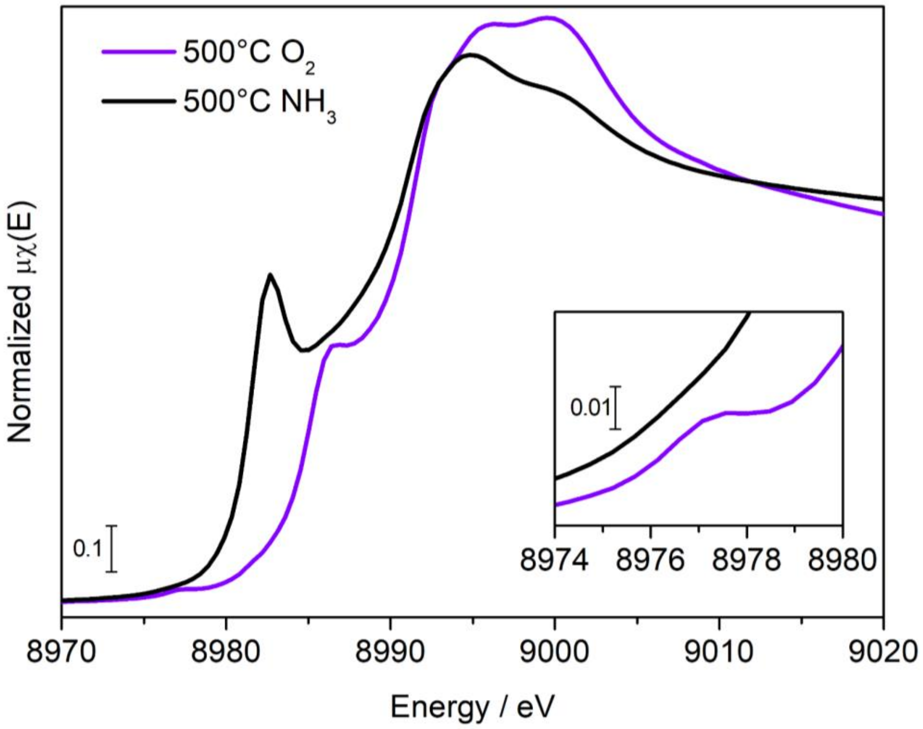

Figure S2. XANES spectra of the (0.45)Cu-MFI(11.5) material after exposure to O_2 and NH_3 at 500°C.

3. Details on the mixed ligand NH_3/CO complex from IR spectroscopy

Figure S3 shows the IR spectrum of the (0.48)Cu-MFI(25) sample after activation and upon interaction with CO in the full acquisition range.

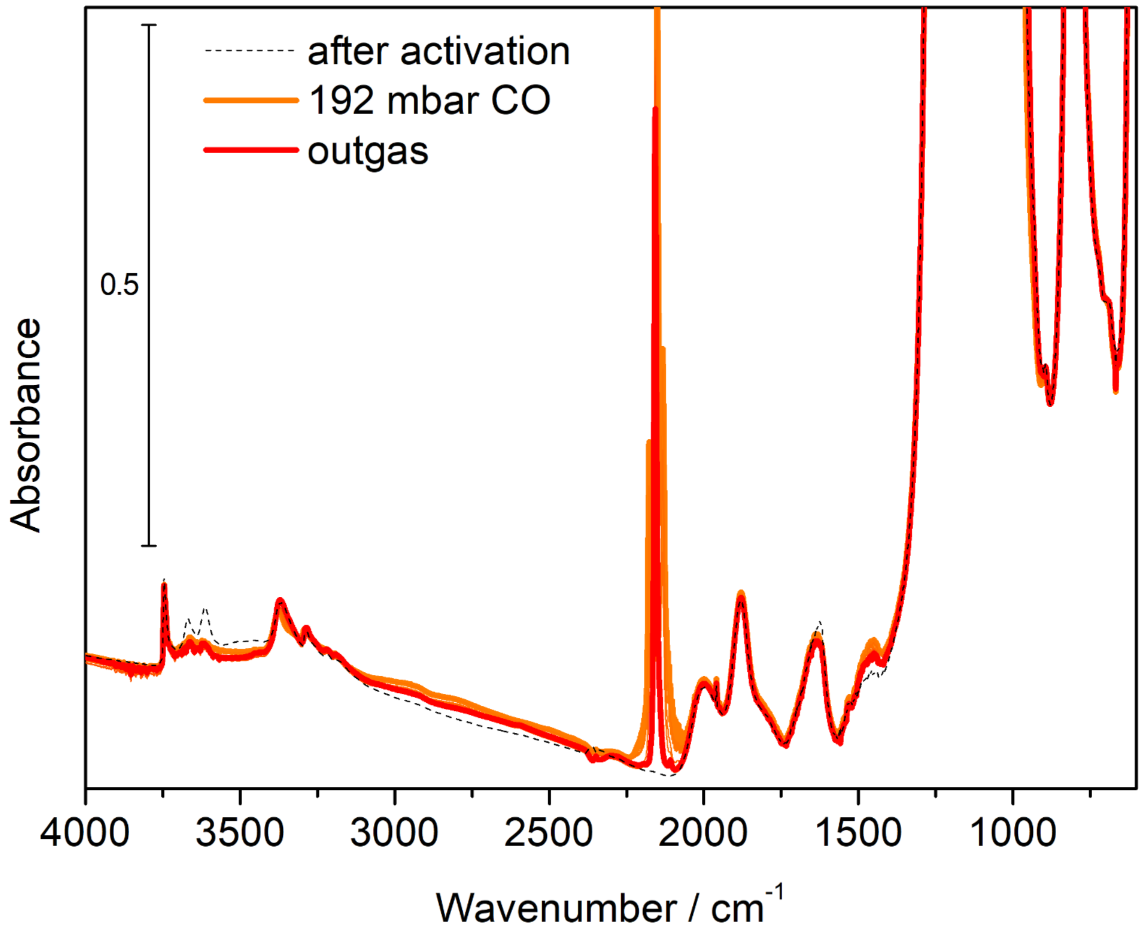

Figure S3. Interaction of CO over the pre-reduced (0.48)Cu-MFI(25) sample.

Traces of adsorbed NH_3 are still present on the sample even after long evacuation at 550°C, as can be seen from the IR spectra of the materials after evacuation. This is suggested by the presence of characteristic bands associated with NH_3 at 3370 and 3280 cm^{-1} (***Figure S4a***)[4]. The residual NH_3 on the material is most probably ligating part of the Cu in the sample; this can be inferred by the IR spectra of the material in the low frequency zone upon interaction with CO, as exemplified for (0.48)Cu-MFI(25) in ***Figure S4b***.

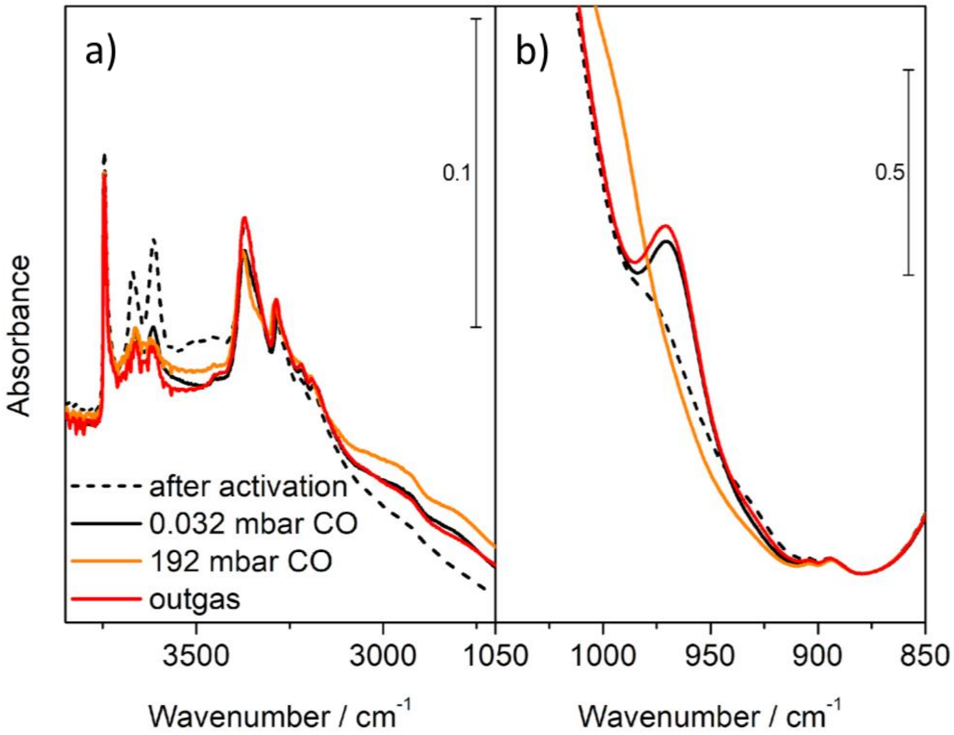

Figure S4. Interaction of CO over the pre-reduced (0.48)Cu-MFI(25) sample. a) Effect of CO on the OH stretching modes. b) Effect of CO on the perturbation of framework modes by Cu.

The band at 2133 cm^{-1} only forms in a relevant amount for the (0.48)Cu-MFI(25) sample (***Figure S5***); this is probably due to the very scarce amount of Brønsted acid sites present in this material compared to the others. The relatively high amount of Cu in this sample could retain an amount of NH_3 that cannot migrate to nearby proton sites, effectively shifting the equilibrium towards a NH_3/CO mixed ligand complex for a higher fraction of the Cu sites.

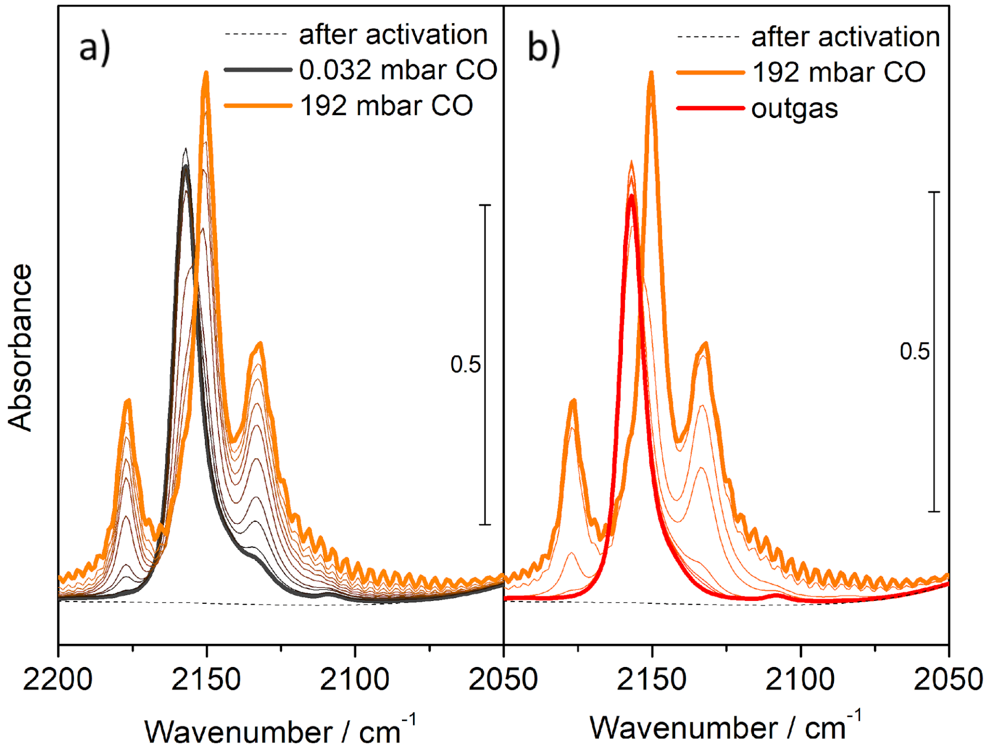

Figure S5. Interaction of CO over the pre-reduced (0.48)Cu-MFI(25) sample. a) Effect of increasing pressure of CO (from black to orange). b) Effect of outgassing on the sample exposed to 192 mbar of CO (from orange to red).

4. CO adsorption isotherms

Figure S6 shows the complete dataset of isotherms collected for the five materials characterized in this paper.

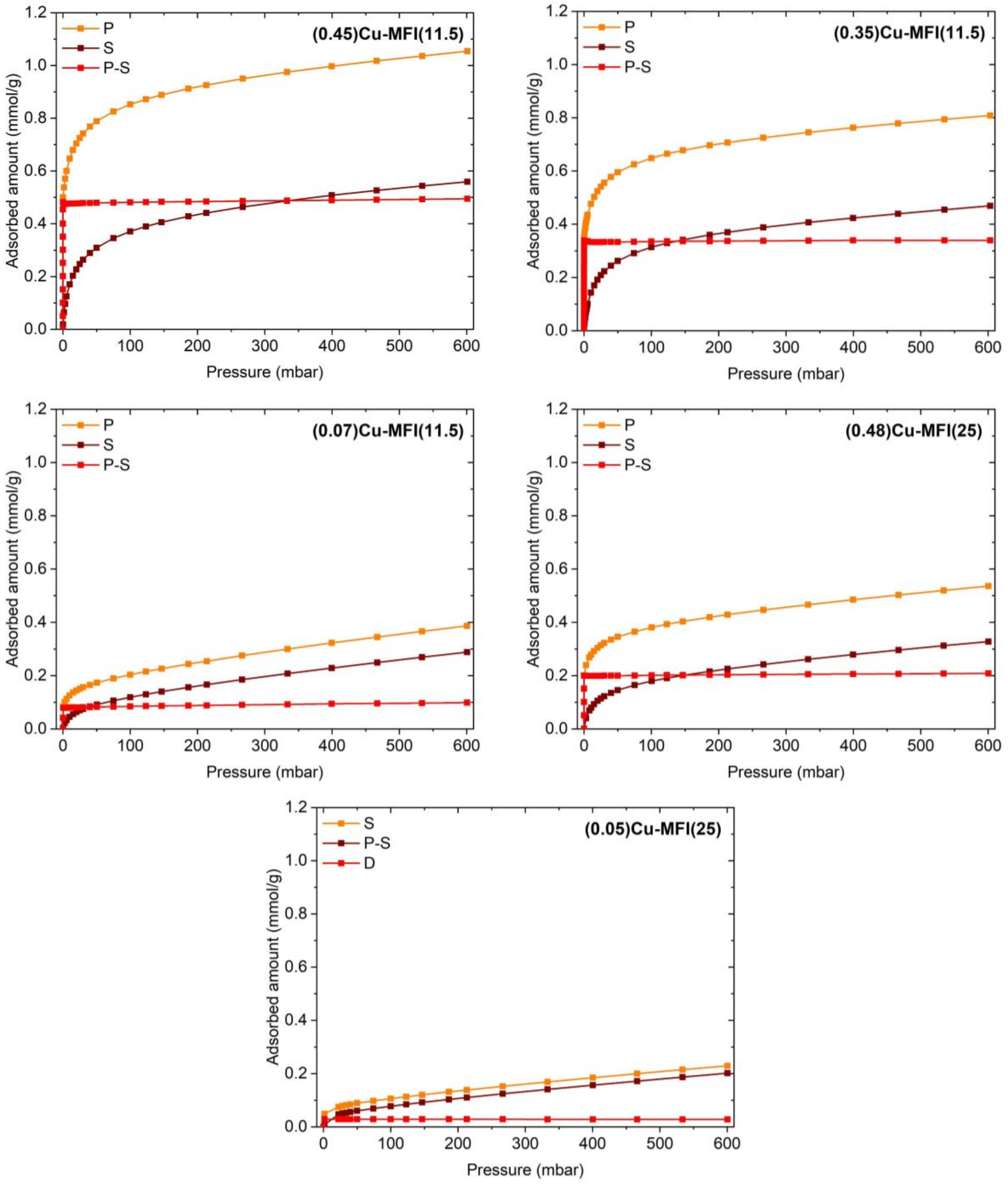

Figure S6. CO adsorption isotherms performed at 50°C on the pre-reduced Cu-MFI samples. Orange: primary isotherm (P). Brown: secondary isotherm (S). Red: difference between the primary and secondary isotherm (P-S), used to calculate the amount of irreversibly bound CO.

5. DFT optimized structures

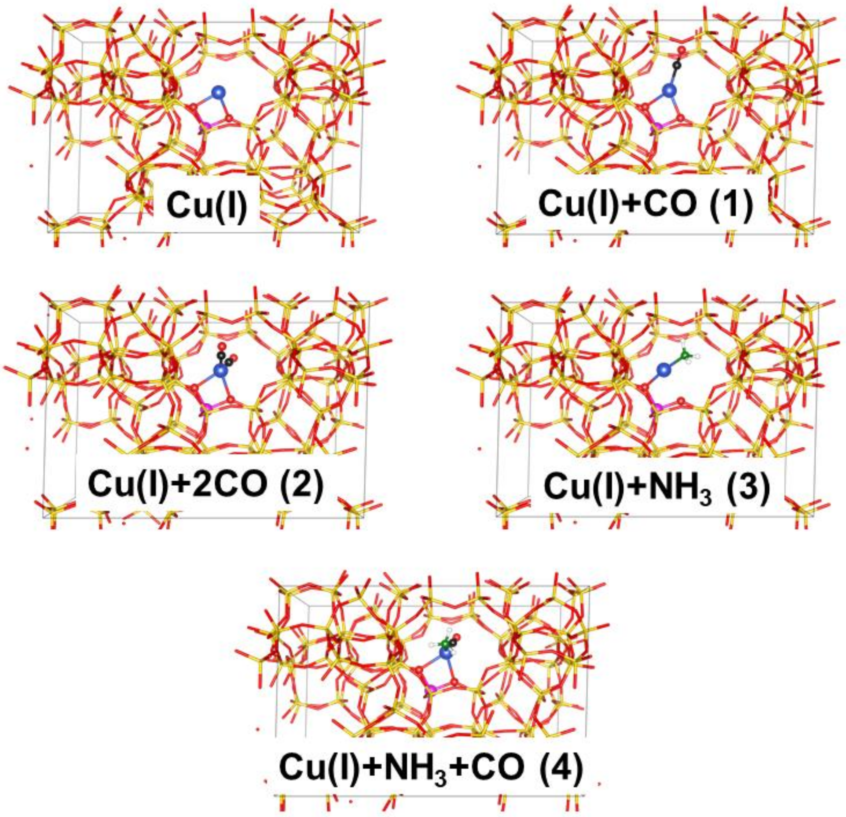

Figure S7. Graphical representation of DFT optimised periodic models of Cu(I) adducts. Atoms colour code: H white; C black; N green; O red; Al purple; Si yellow; Cu blue.

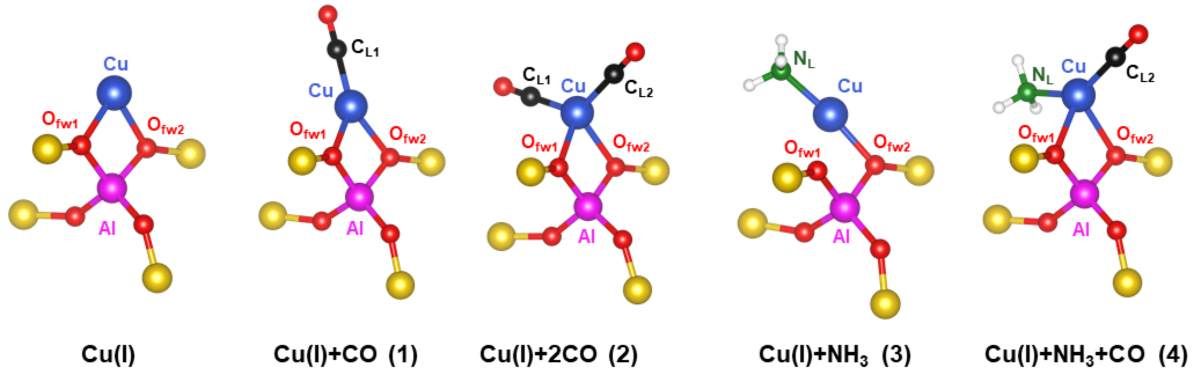

Figure S8. Graphical representation of local structure around Cu for DFT optimised periodic models of Cu(I) adducts. Atoms colour code: H white; C black; N green; O red; Al purple; Si yellow; Cu blue.

Model	a	b	c	α	β	γ	V	Cu-O_{fw1}	Cu-O_{fw2}	Cu-Al	Cu-C_{L1}	Cu-C_{L2}	Cu-N_L
Cu(I)	19.576	19.883	13.286	89.7	91.5	89.9	5169	2.040	2.052	2.755	-	-	-
Cu(I) + CO (**1**)	19.563	19.883	13.284	89.7	91.5	89.8	5165	2.053	2.063	2.792	1.839	-	-
Cu(I) + 2CO (**2**)	19.564	19.880	13.283	89.7	91.5	89.9	5164	2.135	2.1625	2.876	1.930	1.932	-
Cu(I) + NH_3 (**3**)	19.561	19.874	13.279	89.7	91.4	89.8	5161	2.501	1.963	2.823	-	-	1.952
Cu(I) + NH_3 + CO (**4**)	19.568	19.879	13.280	89.8	91.4	89.6	5164	2.188	2.290	2.952	1.851	-	2.046

Table S1. Main geometrical parameters for DFT optimised periodic models of Cu(I) adducts. Cell parameters (a, b, c) and Cu-X distances (α, β, γ) are given in Å, angles in °, volume (V) in Å^3.

Cu environment (in terms of distance to neighbouring O and C atoms) changes upon adsorption with a relative trend that follows what has been previously reported in the literature for similar materials[5]

6. Normalization of the IR spectra of the materials interacting with CO

The spectrum of each material after interaction with CO and outgassing has been arbitrarily shifted to 0 in correspondence with the valley at 1745 cm^{-1}; this point was chosen as the best compromise between minimal interference from scattering effects and minimum absorption from the sample or other molecules. The spectra were then opportunely scaled to a value of absorbance 0.33647 (i.e., the value for one of the samples) at the maximum of the framework modes combination band at 1880 cm^{-1}. This normalization should account for differences in the thickness of the pellets used for the four materials, which were prepared with the intention of keeping the monocarbonyl band in the 0.3-1.0 range for absorbance. Finally, the corresponding spectrum of the activated sample prior to exposure to CO was subtracted to each of them to take scattering effects into account. Integration was performed on the normalized spectra in the 2180-2120 cm^{-1} range.

7. Details on the statistical analysis for the spectrochemical linear model

As mentioned in the main text, the integrated molar attenuation coefficient for the Cu(I)-monocarbonyl stretching peak has been calculated by determining the slope of the equation:

$$A(cm^{-1}) = \varepsilon\left(\frac{cm}{\mu mol}\right)\frac{C(\frac{\mu mol}{g})w(g)}{S(cm^2)}$$

This equation is derived from the Beer-Lambert law, and in this form it does not present an intercept. General linear least-squares regression normally estimates both a slope and an intercept in the fitting procedure, as shown in ***Figure S9***.

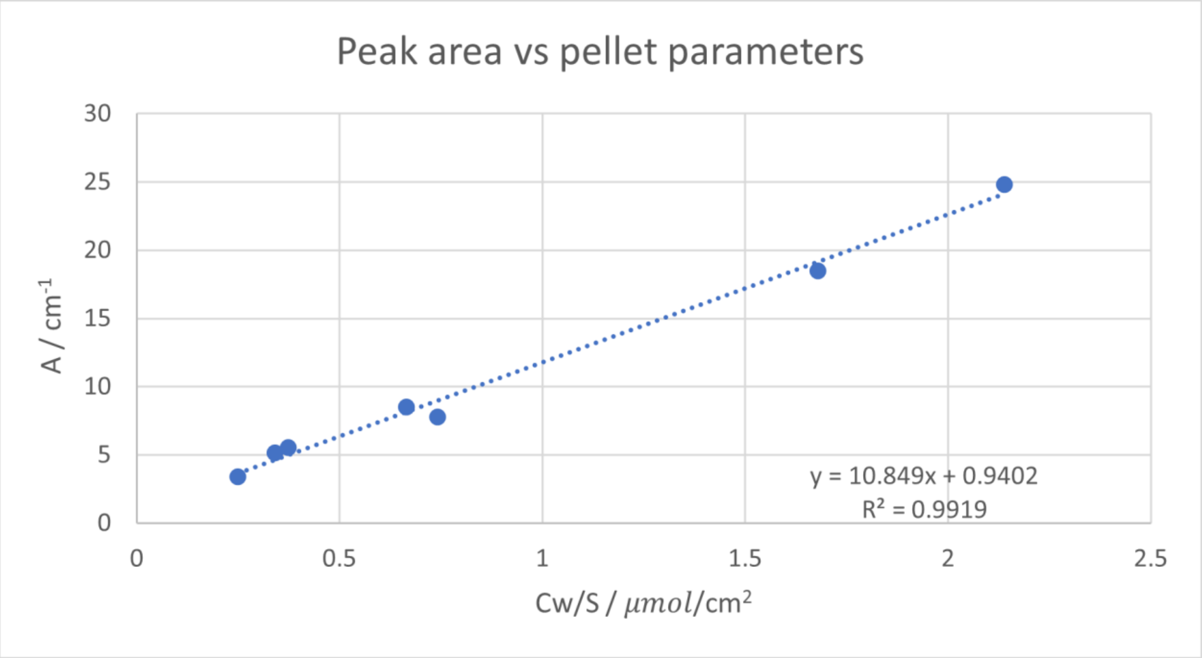

Figure S9. Linear model for quantifying the molar attenuation coefficient relative to the Cu(I) monocarbonyl adducts. For the (0.07)Cu-MFI(11.5), (0.35)Cu-MFI(11.5) and (0.48)Cu-MFI(25) samples two replicas were measured.

A simple Student's t-test, however, suggests that the intercept of this model is statistically equivalent to 0; for this reason, the model presented in ***Figure 4*** is the one that has been used to calculate the slope. This methodology is the one suggested by the NIST handbook for linear calibration[6]. Finally, ***Figure S10*** reports the Residual Plot for the model shown in ***Figure 4***.

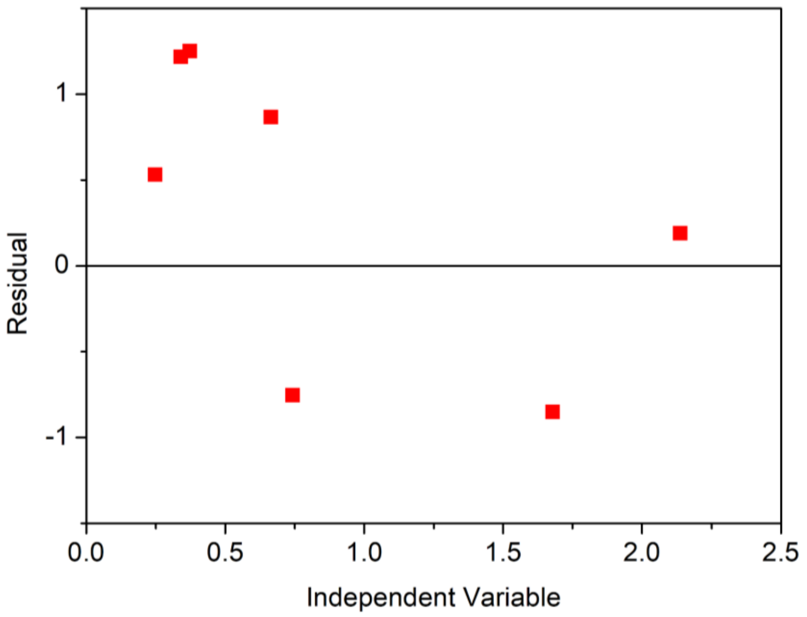

Figure S10. Residual plot for the model presented in Figure 4 (main text).

As can be noticed, the residuals are distributed in a somewhat random manner, and their mean absolute value does not appear to be increasing or decreasing with the independent variable. Thus, the homoscedasticity of the model, together with a good Pearson correlation coefficient (R^2=0.9953), confirms the overall robustness of the model in the explored range for these samples.

References

(1) Ravel, B.; Newville, M. ATHENA, ARTEMIS, HEPHAESTUS: Data Analysis for X-Ray Absorption Spectroscopy Using IFEFFIT. *J. Synchrotron Radiat.* **2005**, *12* (4), 537–541. https://doi.org/10.1107/S0909049505012719.

(2) Groothaert, M. H.; Lievens, K.; van Bokhoven, J. A.; Battiston, A. A.; Weckhuysen, B. M.; Pierloot, K.; Schoonheydt, R. A. Bis(μ-Oxo)Dicopper as Key Intermediate in the Catalytic Decomposition of Nitric Oxide. *ChemPhysChem* **2003**, *4* (6), 626–630. https://doi.org/https://doi.org/10.1002/cphc.200300746.

(3) Borfecchia, E.; Negri, C.; Lomachenko, K. A.; Lamberti, C.; Janssens, T. V. W.; Berlier, G. Temperature-Dependent Dynamics of NH3-Derived Cu Species in the Cu-CHA SCR Catalyst. *React. Chem. Eng.* **2019**, *4* (6), 1067–1080. https://doi.org/10.1039/C8RE00322J.

(4) Zecchina, A.; Marchese, L.; Bordiga, S.; Pazè, C.; Gianotti, E. Vibrational Spectroscopy of NH4+ Ions in Zeolitic Materials: An IR Study. *J. Phys. Chem. B* **1997**, *101* (48), 10128–10135. https://doi.org/10.1021/jp9717554.

(5) Lamberti, C.; Palomino, G. T.; Bordiga, S.; Berlier, G.; Acapito, F. D.; Zecchina, A. Structure of Homoleptic CuI(CO)3 Cations in CuI-Exchanged ZSM-5 Zeolite: An X-Ray Absorption Study. *Angew. Chem. Int. Ed.* **2000**, No. 12, 2138–2141.

(6) https://www.itl.nist.gov/div898/handbook/mpc/section3/mpc361.htm.
